# Safety and Efficacy of Ferric Carboxymaltose in Anemic Pregnant Women: A Retrospective Case Control Study

**DOI:** 10.1155/2015/728952

**Published:** 2015-11-24

**Authors:** Anouk Pels, Wessel Ganzevoort

**Affiliations:** Department of Obstetrics and Gynecology, Academisch Medisch Centrum, Meibergdreef 9, 1105 AZ Amsterdam, Netherlands

## Abstract

*Background*. Anemia during pregnancy is commonly caused by iron deficiency and can have severe consequences for both the mother and the developing fetus. The aim of this retrospective study was to assess the safety and efficacy of intravenous ferric carboxymaltose (FCM) in pregnant women.* Methods*. All women treated with FCM for anemia during pregnancy between 2010 and 2012 at our institution were included. A matched control group was selected, including women who either were nonanemic or had anemia but were not considered for intravenous iron. Main outcome measures were maternal safety and pregnancy outcomes.* Results*. The study included 128 patients (FCM: 64; control: 64). Median FCM dose was 1000 mg and median gestational age at the time of first treatment was 34 weeks and 6 days. Median Hb increased from 8.4 g/dL (interquartile range 7.7; 8.9 g/dL) at the first FCM administration to 10.7 g/dL (9.8; 11.5 g/dL; *n* = 46 with available Hb at delivery) at the time of delivery, achieving levels similar to those in the control group (10.8 g/dL [9.8; 11.8 g/dL; *n* = 48]). No treatment-related adverse events were reported and no statistically significant differences in pregnancy outcomes were observed between groups.* Conclusions*. Within the limitations of this case control study, FCM was a safe and efficient treatment of anemia during pregnancy.

## 1. Introduction

Iron deficiency anemia is a prevalent condition during pregnancy and may result from different factors [1]. Many women have low or empty iron stores already at the start of pregnancy. A large French study, which included a total of 6648 women, showed depleted iron stores (serum ferritin <15 *μ*g/L) in one out of five women (22.7%) of childbearing age [2]. During pregnancy, the physiological need for absorbed iron increases from 0.8 mg/day in the first trimester to 7.5 mg/day in the third trimester [3]. Dietary iron intake does not compensate for this strongly increased iron demand. Consequently, the risk of iron deficiency and, ultimately, iron deficient anemia increases during pregnancy.

General symptoms of anemia are fatigue, dizziness, and impaired immune response predisposing to infections [4]. Anemia during pregnancy is associated with increased morbidity and mortality of pregnant women and their developing fetuses [5]. Iron deficiency anemia has been shown to be associated with an increased risk of premature birth and low birth weight [6], preeclampsia [7], placental abruption, and increased peripartum blood loss [8] as well as cardiac failure and related death [9–11].

In pregnant women, oral iron is often used for prophylaxis of iron deficiency and is recommended as first-line treatment for pregnant women with iron deficiency anemia [12]. However, oral iron substitution has shown to be insufficient for the treatment of severe iron deficiency anemia and is often associated with gastrointestinal side effects [13]. Therefore, guidelines recommend that physicians consider intravenous (i.v.) iron administration in pregnant women with severe iron deficiency anemia (Hb < 9.0 g/dL), and in case of intolerability to oral iron as well, insufficient Hb increase after oral iron treatment or if there is a need for rapid Hb reconstitution [12–14]. Intravenous (i.v.) iron preparations provide greater and more rapid repletion of iron stores than oral iron therapy without the gastrointestinal side effects associated with oral substitution [13].

Ferric carboxymaltose (FCM) is an i.v. iron formulation which can be used at high doses and allows rapid administration (up to 1000 mg in a single dose infused in 15 min). Because it is free of dextran and its derivatives, FCM does not cross-react with dextran antibodies [15, 16] and never needed the administration of a test dose. More recently, the European Medicines Agency (EMA) concluded that no test dose should apply to i.v. iron products authorized in the European Union; yet staff and facilities to evaluate and manage anaphylactic or anaphylactoid reactions should be immediately available [17]. The FCM molecules consist of an iron-hydroxide core chelated in a carbohydrate shell and this complex is taken up as a whole by macrophages, leading to very low levels of non-transferrin bound iron, avoiding iron toxicity and oxidative stress [16]. FCM's clinical efficacy and safety have been proven in several large clinical studies across different indications with up to one-year follow-up in severe disease types such as chronic kidney disease and chronic heart failure [18–28]. At least four postpartum studies compared the safety and efficacy of FCM versus oral iron [26–29]. Faster and greater Hb-responses were achieved in FCM-treated patients compared to those receiving oral iron and FCM replenished iron stores efficiently. Rather few studies or cases with limited numbers of FCM-treated pregnant women have been reported [30–33]. A recently completed study comparing FCM and oral iron in pregnant women with iron deficiency anemia (ClinicalTrials.gov NCT01131624) has not been reported yet.

The aim of this retrospective case control study was to assess the efficacy of i.v. FCM in pregnant women.

## 2. Materials and Methods

### 2.1. Study Design and Patients

Data for this retrospective case control study were obtained from the electronic patient charts of the Department of Obstetrics and Gynecology of the Academisch Medisch Centrum in Amsterdam, Netherlands. The study design has been reviewed by the Medical Ethical Committee of the Academisch Medisch Centrum and it was confirmed that an official approval of this study by the committee is not required, since the Medical Research Involving Human Subjects Act (WMO) does not apply.

Patients were identified by searching the digital records of the Department of Obstetrics and Gynecology for women who received FCM (Ferinject, Vifor Pharma Ltd., Switzerland) treatment and/or delivered a baby between 2010 and 2012. All women who received at least one administration of FCM during their pregnancy were eligible for the case group. Women who were treated with FCM in the postpartum period were excluded from the analysis group, but safety data were collected. The control group was formed by an equal number of pregnant women who either were nonanemic or had anemia to a lesser degree not necessitating i.v. iron treatment. The women in the control group were matched to the case group for delivery period, type of comorbidity, age, parity, and number of fetuses.

### 2.2. Treatment Characteristics

Pregnant patients with anemia were treated according to the local protocol. The institutional anemia cutoff value throughout advanced gestation is approximately 9.7 g/dL (1.0 g/dL = 0.62 mmol/L). According to the local protocol, pregnant women with anemia are treated with oral iron (ferrous fumarate, one 200 mg tablet per day) and switched to i.v. iron, if Hb remains <9.7 g/dL despite oral medication. FCM is the institution's first choice i.v. iron agent since 2010, regardless of iron status. The local protocol does not describe the time frame in which the Hb should increase to above 9.7 g/dL. The maximum weekly dose of FCM is 1000 mg (up to 20 mg/kg body weight) in a single infusion given over at least 15 minutes.

### 2.3. Outcome Measures

Demographic characteristics and baseline data included maternal age, gestational age, educational level, and results from peripheral blood counts. Outcome data were collected on adverse events and pregnancy outcomes. Adverse events (AEs) in FCM-treated patients were defined as allergic or hypersensitivity reactions during or after the infusion of FCM. Assessed pregnancy outcomes were hospital admission (before delivery, for other reasons than FCM administration), intensive care unit admission, intrauterine growth restriction (IUGR), hypertension/preeclampsia, placental abruption, major adverse outcomes (maternal or fetal), minor maternal adverse outcomes, Hb at delivery (g/dL), need for red blood cell transfusion, gestational age at delivery, mode of delivery, estimated blood loss during delivery, fetal weight (g), and neonatal Apgar score.

Major maternal adverse outcomes were defined as death, stroke, neurological symptoms, severe preeclampsia, HELLP (Hemolysis Elevated Liver enzymes Low Platelets) syndrome, and delivery before 34 weeks of gestation. Major adverse fetal outcomes were defined as death, respiratory problems (requiring intubation), neonatal intensive care unit admission, pneumonia, morbidity requiring surgery, birth problems, and Apgar score <7.

The charts were liberally screened for minor adverse outcomes with an inclusive strategy, to include a variety of nonprespecified events.

The local protocol for iron treatment requires minimal diagnostics and follow-up assessment of hematologic iron status parameters. For this reason, Hb measurements and Mean Corpuscular Volume (MCV) were recorded at FCM treatment (in the case group) and Hb measurements at delivery (in case and control group).

### 2.4. Statistical Analysis

No formal sample size calculation was made, since all FCM-treated women who fulfilled the inclusion criteria were included. Statistical analysis was performed using IBM SPSS Statistics. Safety and efficacy were analyzed using descriptive statistics, comparing the case group to the control group. For the case group, medians have been calculated for the dose, the gestational age at treatment, and Hb at treatment with FCM. For statistical comparison, *t*-test and Chi-Square test were used, where appropriate. *p* values < 0.05 were considered statistically significant.

## 3. Results

We identified 85 women who received FCM between 2010 and 2012 at our institution. Three women were excluded from the study, since they were not pregnant during the treatment with FCM, and 18 women received FCM postpartum. Sixty-four women received at least one FCM administration during pregnancy and were included in the case group. These patients were matched with 64 controls.

Demographic characteristics such as age (median: 27 years versus 28 years), parity (median 1 versus 1), number of fetuses (singleton pregnancies: 92% versus 92%), percentage of patients with lower educations (20% versus 16%), and prevalence of comorbidities (20% versus 19%) were similar between case and control groups (Table 1). Individual comorbidities were present at low frequency and none of the comorbidities was dominant (1-2 patients per comorbidity). Of note, there were significantly more women of Caucasian origin in the control group (34% versus 7.8%) and more patients were affected by a familial disease in the case group (59% versus 38%). The most frequent familial disease was concomitant hypertension and diabetes (28% in the case group and 20% in the control group). No information about dietary habits or other lifestyle interventions was recorded.

The median FCM dose in the case group was 1000 mg (interquartile range [IQR]: 1000; 1500) and most women (51/64) received only one dose of FCM (Table 2). The maximum number of administered FCM treatments was three (in 3 out of 64 patients) due to persistent anemia. Median gestational age at the time of the first treatment was 244 days (IQR 224; 256 days). Median Hb at first FCM administration was 8.4 g/dL (IQR: 7.7; 8.9 g/dL) and increased to 10.7 g/dL (IQR: 9.8; 11.5 g/dL) at the time of delivery, achieving a similar level as in the control group (10.8 g/dL [IQR: 9.8; 11.8 g/dL]) (Table 3).

No treatment-related adverse events were reported in the case group. Of note, FCM was also used by 18 women in the postpartum period, mostly after postpartum hemorrhage. These women were not included in the case group but their charts were reviewed for adverse events related to the treatment with FCM. In the women who used FCM postpartum no serious adverse events (allergic or hypersensitivity reactions) were reported.

No statistically significant differences were seen in the pregnancy outcomes between groups (Table 3). Major adverse outcomes in the case group were delivery before 34 weeks of gestation (*n* = 5), death of the fetus (*n* = 3), atrioventricular septal defect (*n* = 1), respiratory problems (*n* = 1), pneumonia and skin abnormalities (*n* = 1), and Apgar score <7 (*n* = 1). Major adverse outcomes in the control group were severe preeclampsia (*n* = 1), HELLP syndrome (*n* = 3), encephalopathy with hepatic dysfunction (*n* = 1), delivery before 34 weeks of gestation (*n* = 5), death of the fetus (*n* = 3), neonatal intensive care unit admission (*n* = 2), respiratory problems (*n* = 1), jejunum resection (*n* = 1), severe shoulder dystocia (*n* = 1), tachycardia, low saturation and hepatomegaly (*n* = 1), and Apgar score <7 (*n* = 2).

## 4. Discussion

This case control study investigated the efficacy and safety of FCM during pregnancy. FCM treatment efficiently increased Hb in the case group from baseline to delivery to similar levels as were present in the control group. There were no hypersensitivity reactions, anaphylactic reactions, or other adverse events reported with FCM treatment, neither in the case group nor in the group who received FCM postpartum. Maternal and fetal outcomes were similar between the case and the control group.

Most women received only a single dose of 1000 mg iron which is in line with the institution's protocol. Only a minority of women required more than one FCM administration due to persistent anemia (maximum of three iron administrations in three women). This practice of single dose administration is facilitated by the greater stability of the FCM complex compared to less stable i.v. iron compounds such as ferric gluconate and iron sucrose that require multiple administrations of lower doses.

Our results are in line with a number of randomized controlled studies which have shown the safe and efficient use of FCM in the field of gynecology during the postpartum period [26–29] and in heavy uterine bleeding [22]. Randomized controlled studies on the use of FCM during pregnancy are, to date, lacking. In a retrospective study, Christoph et al. [30] showed that, in the second and third trimester, FCM was equally well tolerated as i.v. iron sucrose and demonstrated comparable rates of adverse events (8% FCM; 11% iron sucrose). All adverse events were classified as mild and quickly reversible and included local reactions (such as pain and rash at the injection site) and systemic effects (such as transient hypotension, dizziness, heart palpitation, nausea, and headache). However, the two groups of pregnant women were heterogeneous in i.v. iron treatment indication and therefore not really comparable, regarding pregnancy complications. A study by Myers et al. [31] comparing FCM to iron hydroxide dextran treatment showed similar results regarding adverse events and treatment effect but no pregnancy outcomes were reported. Froessler et al. [32] performed a prospective observational study including 65 anemic pregnant women who received FCM. In this patient group, Hb levels significantly increased after FCM treatment and 66% of women reported an improvement of their well-being after the infusion. No serious adverse effects were reported. Minor side effects occurred in 13 (20%) patients and were self-limiting except for one case of nausea and vomiting which required medication. Froessler et al. also published two case reports of anemia successfully treated using ferric carboxymaltose in the peripartum period [33].

There were some noteworthy differences in the baseline characteristics between the case and the control group in our study. Firstly, the case group contained more women of ethnic minorities which generally have a lower socioeconomic status and worse general health than women of Caucasian ethnicity in our population. Secondly, the case group contained more women with familial hypertension/diabetes than the control group. In addition, women in the control group either were nonanemic or had anemia to a lesser degree than the case group. The expected direction of bias caused by these described differences between case and control group would be to the disadvantage of the case group. In spite of these apparent disadvantages in the case group, we found that pregnancy outcomes were similar in case and control group.

Due to the retrospective case control design, our study has some limitations. Choosing adequate controls in a case control design is difficult. For the control group, we could also have chosen women with a similar degree of anemia as the case group, receiving either no or alternative medication, but these groups are not easily identified and comparisons are subject to indication bias. Due to the retrospective data collection, not all of the assessed parameters were available for all patients and consequently, numbers/medians of certain parameters were based on less than 128 patients (e.g., ethnicity, level of education, familial diseases, and Hb at delivery). Data on additional iron status parameters such as serum ferritin (indicating stored iron) or transferring saturation (indicating iron available for erythropoiesis) would have been useful but these were not required by the local protocol.

While we would not expect any serious events in our case group due to the low patient number (*n* = 64) and the rarity of such events (life threatening adverse events related to i.v. iron preparations generally occur at a rate of 0.6–11 per million patients depending on the i.v. iron compound [31]), we suspect that mild and transient adverse events were underreported in the analyzed patient charts.

## 5. Conclusions

In conclusion, FCM was effective in treating anemia in this population of pregnant women in the 3rd trimester and appears to be safe for mother and child, although no definite conclusion about safety can be drawn from the results of this small case group. A prospective randomized controlled trial is warranted for a more detailed analysis on pregnancy outcomes.

## Figures and Tables

**Table 1:** Demographics.

Characteristics^*∗*^	Case group (*n* = 64)	Control group (*n* = 64)	*p* value
Age, years	27 [17–39]	28 [17–40]	0.71
Parity, *n*	1 [0–4]	1 [0–4]	0.87
Ethnicity			
Caucasian	5 (8%)	22 (34%)	0.00
African descent	38 (59%)	15 (23%)	
Other	11 (17%)	16 (25%)	
Unknown	10 (16%)	11 (17%)	
Education level			
Lower education	13 (20%)	10 (16%)	0.18
Middle education	21 (33%)	12 (19%)	
Higher education	7 (11%)	12 (19%)	
Unknown	23 (36%)	29 (46%)	
Comorbidities^†^			
Overall	13 (20%)	12 (19%)	0.12
Preexisting hypertension	1 (2%)	2 (3%)	
Preexisting diabetes	1 (2%)	2 (3%)	
Renal/liver transplant	1 (2%)	0	
Renal malignancy in the past	1 (2%)	1 (2%)	
Irritable bowel syndrome	0	1 (2%)	
Uterus myomatosus	2 (3%)	1 (2%)	
Primary hyperoxaluria type 1	1 (2%)	0	
Asthma	1 (2%)	1 (2%)	
Hypothyroidism	2 (3%)	1 (2%)	
Sickle cell anemia	2 (3%)	1 (2%)	
Rheumatoid arthritis	1 (2%)	0	
Ehlers-Danlos type III	0	1 (2%)	
Alpha thalassemia	0	1 (2%)	
HIV infection	1 (2%)	1 (2%)	
IL-12 receptor deficiency	0	1 (2%)	
Unknown	4	0	
Familial diseases			
None	14 (22%)	36 (56%)	0.00
Hypertension	14 (22%)	8 (13%)	
Diabetes	6 (9%)	3 (5%)	
Hypertension and diabetes	18 (28%)	13 (20%)	
Unknown	12 (19%)	4 (6%)	
Number of fetuses			
Singleton pregnancy	59 (92%)	59 (92%)	1.00
Twin pregnancy	5 (8%)	5 (8%)	

^*∗*^Data shown as median [range] or *n* (%); ^†^patients could have more than one comorbidity.

**Table 2:** Treatment characteristics of the case group (*n* = 64) treated with FCM.

Treatment characteristics^*∗*^	
Hb at 1st FCM treatment, g/dL (*n* = 62)^†^	8.4 [7.7; 8.9]
Hb at 1st FCM treatment, mmol/L (*n* = 62)^†^	5.2 [4.8; 5.5]
MCV at 1st FCM treatment, fL (*n* = 49)^†^	69 [62; 76]
Gestational age at 1st treatment, weeks (*n* = 64)^‡^	34 + 6 [32; 36 + 4]
Gestational age at 2nd treatment, weeks (*n* = 13)^‡^	35 + 2 [32 + 6; 37 + 3]
Gestational age at 3rd treatment, weeks (*n* = 3)^‡^	32 [N/E]
Total dose received, mg	1000 [1000; 1500]
Treatment-related adverse outcomes reported	0 (0%)
Treatment-related serious adverse outcomes	0 (0%)

^*∗*^Data shown as median [IQR] or *n* (%); ^†^patients with available data (for remainder of study population not reported); ^‡^total number of patients receiving treatment; Hb: hemoglobin; MCV: mean corpuscular volume; IQR: interquartile range.

**Table 3:** Pregnancy outcomes in cases and controls.

Pregnancy outcome^*∗*^	Case group (*n* = 64)	Control group (*n* = 64)	*p* value
Hospital admission (*n* = 126)^†^	27 (42%)	20 (31%)	0.13
ICU admission (*n* = 127)^†^	1 (2%)	2 (3%)	0.51
IUGR (*n* = 127)^†^	2 (3%)	5 (8%)	0.30
Hypertension/preeclampsia (*n* = 126)^†^	8 (13%)	9 (14%)	0.36
Placental abruption (*n* = 126)^†^	1 (2%)	0 (0%)	0.22
Median Hb at delivery, g/dL (*n* = 94)^†^	10.7 [9.8; 11.5]	10.8 [9.8; 11.8]	0.76
Median Hb at delivery, mmol/L (*n* = 94)^†^	6.7 [6.1; 7.1]	6.7 [6.1; 7.3]	0.76
Unknown Hb at delivery	18 (28%)	16 (25%)	
Major adverse outcome (*n* = 128)^†^			
Maternal only	3 (5%)	4 (6%)	0.50
Fetal only	5 (8%)	5 (8%)	
Maternal and fetal	2 (3%)	6 (9%)	
Minor maternal outcome^‡^ (*n* = 127)^†^	23 (36%)	18 (28%)	0.36
Need for transfusion of blood products (*n* = 125)^†^	2 (3%)	3 (5%)	0.20
Gestational age at delivery, weeks (*n* = 124)^†^	39.2 [38.0; 40.3]	39.1 [36.7; 39.9]	0.18
Mode of delivery (*n* = 126)^†^			
Spontaneous vaginal	46 (72%)	39 (61%)	0.29
Assisted vaginal	2 (3%)	5 (8%)	
Primary Caesarean	9 (14%)	12 (19%)	
Secondary Caesarean	*5 (8%)*	*8 (13%)*	
Blood loss during delivery, mL (*n* = 124)^†^	300 [200; 400]	300 [200; 538]	0.64
Fetal weight, g (*n* = 128)^†^			
Singleton babies	3235 [3025; 3565]	3210 [2710; 3500]	0.64
Twin babies	2400 [1872; 2721]	2343 [1675; 2618]	0.33
Apgar score (*n* = 125)^†^			
Singleton babies	10 [10; 10]	10 [9; 10]	0.73
Twin babies	9 [7; 10]	9 [7; 10]	0.92

^*∗*^Data shown as median [IQR] or *n* (%); ^†^patients with available data (for remainder of study population not reported/unknown).

^‡^Minor maternal outcomes: malaise (2 in case group), abdominal pain (3 in case group, 2 in control group), nausea and vomiting of pregnancy (NVP) (3 in case group, 3 in control group), premature contractions (3 in case group), Preterm Premature Rupture Of Membranes (PPROM) (4 in case group, 5 in control group), impaired renal function (1 in case group), meconium amniotic (1 in case group), vaginal blood loss (1 in case group, 1 in control group), preeclampsia (3 in case group), hypertension (2 in case group, 3 in control group), severe hypertension (1 in case group), postpartum hypertension (1 in control group), fever (2 in case group), postpartum fever (2 in control group), urinary tract infection (2 in case group), trauma (1 in case group), reduced signs of fetal life (2 in case group), back pain (1 in case group), tachycardia (1 in case group), gestational diabetes (2 in case group, 1 in control group), herpes gestationis (1 in case group), gestational pyelitis (1 in control group), pulmonary embolism (1 in control group), and positive discongruence (1 in control group).

Hb: hemoglobin; IQR: interquartile range; ICU: intensive care unit; IUGR: intrauterine growth retardation.
